# Neurocognition and social cognition in remitted first-episode schizophrenia: correlation with VEGF serum levels

**DOI:** 10.1186/s12888-019-2397-8

**Published:** 2019-12-16

**Authors:** Yaqin Zhao, Wenhuan Xiao, Kuanyu Chen, Qiongqiong Zhan, Fei Ye, Xiaowei Tang, Xiaobin Zhang

**Affiliations:** 1 Department of Psychiatry, Affiliated WuTaiShan Hospital of Medical College of Yangzhou University, Yangzhou, Jiangsu 225003 People’s Republic of China; 2 Institute of Mental Health, Suzhou Psychiatric Hospital, The Affiliated Guangji Hospital of Soochow University, Suzhou, Jiangsu 215137 People’s Republic of China; 3 School of mental health, Jining medical University, Jining, 272000 Shandong China; 4 Huzhou University, Huzhou, 313000 Zhejiang China; 5 Nanjing Brain Hospital, Nanjing Medical University, Nanjing, 210029 Jiangsu China

**Keywords:** Schizophrenia, Vascular endothelial growth factor, Neurocognition, Social cognition

## Abstract

**Background:**

Accumulating evidence suggests that serum vascular endothelial growth factor (VEGF) in many neurobiological processes potentially contributes to the pathophysiology of psychiatric disorders, particularly cognitive decline. The purpose of this study was to explore the differences in neurocognition, social cognition and VEGF among remitted first-episode schizophrenic patients, non-remitters and normal control subjects. Moreover, we investigated the association between serum VEGF levels and cognitive functions.

**Method:**

65 remission (RS) and 45 nonremission patients (NRS) after first-episode schizophrenia, as well as 58 healthy controls (HC) were enrolled in this study. Social cognition was assessed using the Chinese Facial Emotion Test (CFET); neurocognition was measured with a test battery consisting of Hopkins Verbal Learning Test-Revised, Verbal Fluency Test, Trail Making Tests, Digit Span Tests (DST) and Stroop Tests. Blood samples were collected for VEGF measurements. Data was analyzed with SPSS 22.0 (Chicago, IL, USA).

**Results:**

On nearly all neurocognitive tests (except for DST), RS performed significantly worse than HC but better than NRS (*P <* 0.05). NRS, but not RS, exhibited markedly poorer social cognition than HC (except for Happiness and Surprise subscales of the CFET) (*P* < 0.05). VEGF levels showed a gradient change among three groups (HC > RS > NRS).

**Conclusion:**

Compared to HC, RS demonstrated poorer neurocognitive but intact social cognition functioning. These results indicate that VEGF levels decreased gradually with the severity of cognitive impairment in schizophrenia. VEGF may be involved in the pathological mechanism of cognitive performance in RS.

## Background

Cognitive impairment, a core symptom of schizophrenia, is characterized by neurocognition and social cognition deficits [1, 2]. Recent neuropsychological studies have indicated that cognitive dysfunction occurs in unaffected first-degree relatives of individuals with schizophrenia [3–5], and almost all patients in remission after first-episode schizophrenia have experienced an improvement in cognitive function following treatment with atypical antipsychotics [6, 7]. In contrast, some scholars believe that impairment of cognition including working memory, attention and executive function is independent of psychiatric symptoms, and may be considered as specific and persistent trait markers of schizophrenia [8].

Schizophrenic patients also present with deficits in certain aspects of social cognition [9, 10]. Recent studies showed that social cognition deficit occurred during the early development of mental disorders and persisted until the improvement of clinical symptoms [5, 11, 12], suggesting that social cognition deficit may serve as a vulnerability marker of this disorder. It is noteworthy that these studies included patients with acute episodes, chronic episodes and minor remission. Therefore, it is not known whether this persists during clinical remission in patients with first-episode schizophrenia.

Vascular endothelial growth factor (VEGF) is not only a member of the prominent and well-characterized neurotrophin family, but also an angiogenetic factor [13]. VEGF can protect against brain cell loss, blood–brain barrier dysfunction, dendritic spine loss, spatial memory impairment and cognitive decline in response to injury [14, 15]. In addition, VEGF has been reported to regulate blood flow and induce vasopermeability of vascular endothelial cells [13], and VEGF deficiency contributes to alterations in cellular energy metabolism and blood flow in the regional brain. Therefore, we hypothesized that abnormal expression of VEGF may be involved in the pathological mechanisms underlying hypoperfusion or decreased blood flow observed in patients with schizophrenia.

Animals experiments revealed that VEGF reversibly modulated hippocampal synaptic plasticity and improved hippocampal activity related to learning and memory [13, 16, 17]. Furthermore, a recent study showed that VEGF was upregulated in the parietal cortex of patients with schizophrenia compared to controls, and the levels of VEGF in serum were related to prefrontal cortical volume of schizophrenia subjects [18]. Consistent with the animal experiments, human studies demonstrated that VEGF levels were associated with cognitive function in patients with Alzheimer’s disease [15, 19, 20]. These findings support the notion that VEGF is an important factor against cognitive decline in neuropsychiatric illnesses. However, the effect of VEGF on cognitive function in remitted first-episode schizophrenic patients still remains unclear.

To date, it is not clear whether neurocognition, social cognition and VEGF can be restored close to their normal levels in remitted first-episode schizophrenia, and whether VEGF represents a stable trait marker or a temporary state marker of the illness. Therefore, the purpose of this study was to explore neurocognition and social cognition as well as VEGF levels and their relationship with symptom remission in first-episode schizophrenia. We hypothesized that cognition and VEGF deficits were more pronounced in patients with non-remitted schizophrenia (NRS) as compared to healthy controls (HC) but not patients with remitted schizophrenia (RS), and that these abnormalities persisted even after remission of the patient’s psychiatric symptoms.

## Methods

### Subjects

A total of 110 patients with first-episode schizophrenia (65 RS and 45 NRS) and 58 normal controls were enrolled. Inclusion criteria for patients were: schizophrenia diagnosis (according to DSM-V); first episodes of schizophrenia; aged 18–55 years; All RS were from outpatient clinics and NRS were from both inpatient and outpatient clinics. All patients had been hospitalized at least once at Wutai Mountain Hospital in Yangzhou, Jiangsu Province, China. A total of 95 patients were treated with monotherapy of atypical antipsychotic drugs: clozapine (*n* = 25), olanzapine (*n* = 18), risperidone (*n* = 18), ziprasidone (*n* = 15), quetiapine (*n* = 12) and aripiprazole (*n* = 7); and 15 patients received a combination with other agents, remission: aripiprazole plus clozapine (*n* = 2), non-remission: risperidone plus quetiapine (*n* = 2), ziprasidone plus clozapine (*n* = 2), risperidone plus clozapine (*n* = 4), olanzapine plus aripiprazole (*n* = 3), quetiapine plus aripiprazole (*n* = 2). According to Andreasen [21], any patient who achieved at least a ‘mild or better’ score of eight items (three items on psychoticism, two on disorganization and three on negative symptoms) of the PANSS, and the score maintained for at least 6 months, was considered remitted. Exclusion criteria were: head trauma history, neurological disease, drug abuse, other mental illness related to cognitive manifestation. In addition, demographic and clinical variables such as age, sex, education period, age of illness onset, course of illness, and antipsychotic dose (converted to chlorpromazine equivalent) were collected.

The control group consisted of 58 healthy subjects who volunteered to participate in this trial. All control subjects were in good physical health with normal laboratory findings (renal function, thyroid function, liver function and electrocardiography) and had not a history of mental disorders, assessed by means of The Mini International Neuropsychiatric Interview (MINI) [22], or first-degree relatives with a history of mental disorders or brain disease (e.g. dementia and hypothyroidism) associated with cognitive ability. General information (age, sex, education period, number of smokers and body mass index) was matched between patients and controls.

All participants (or parents/legal guardians) provided signed informed consent prior to participation. The study was approved by the Ethics Committee of Yangzhou University.

### Cognitive measurement

A battery of cognitive tests was administered to all subjects to measure neurocognitive performance: Hopkins Vocabulary Learning Test-Revised (HVLT-R), Verbal Fluency Test (VFT-animals and VFT-actions), Trail Making Tests (TMT-part A and TMT-part B) and Digit Span Tests (DST-Forward and DST-Backward), as well as Stroop Tests (words, colors and interference). Chinese Facial Emotion Test (CFET) was used for assessing social cognition, which includes six emotions (happiness, sadness, fear, disgust, anger, surprise) [23–25]. Cognitive assessment was performed by two experienced psychiatry specialists in the test laboratory and validated by an inter-rater correlation coefficient > 0.8. The administration of cognitive tests took 2 h on average. Higher test scores indicate better cognitive function (except for the TMT). Not all patients were able to complete all cognitive tests, 2 patients in the NRS group did not complete all cognitive tests (one did not complete DST-Backward, the other did not complete Stroop interference).

### VEGF serum analysis

Blood samples were collected in anticoagulant-free tubes during the morning (between 08:00 and 09:00 h). Serum was then separated, sealed and stored at − 80 °C. VEGF levels were measured using a commercially available kit (DVE00; R&D Systems, Minneapolis, IN, USA) according to the manufacturer’s instructions. All tests were conducted in duplicate for each concentration. Calculation of VEGF was performed using a standard curve for recombinant human VEGF protein. The lower detection limit was 9 pg/mL. Intra- and inter-assay variances were below 5.1 and 6.2%, respectively. Sample collection and analyses were performed in a blinded manner.

### Statistical analysis

The Kolmogorov–Smirnov test was performed to test normality of data. For normally distributed continuous variables, Student’s unpaired t-test or ANOVA was used for data comparisons between two or among more groups. Post-hoc analyses were done by multiple comparison tests with Bonferroni corrections. Not normally distributed variables were evaluated with Mann-Whitney or Kruskal–Wallis H tests. Analyses of categorical variables were carried out using a Chi-squared (χ2) test. Spearman’s correlation coefficients were used to analyze the correlation between VEGF and other parameters (demographic and clinical variables, cognitive performance) among the study groups. Partial correlation analysis was employed to explore the potential influence of confounding variables on cognitive performance. Differences of *P* < 0.05 were considered to be significant. Data were analyzed with SPSS 22.0 (Chicago, IL, USA).

## Results

### Socio-demographic variables, clinical characteristics and serum VEGF levels

There was no significant difference in age, sex, education period, number of smokers and body mass index among the three groups (*P* > 0.05). RS and NRS did not differ in the age of onset of schizophrenia and their family history of mental illness (*P* > 0.05). There were no significant differences in the sociodemographic and clinical characteristics and VEGF levels between in and outpatients (*P >* 0.05). However, NRS used a significantly higher antipsychotic drug dose (chlorpromazine equivalent) relative to RS patients (*t* = 3.298, *df* = 108, *P* = 0.001) (Table 1).

**Table 1:** Sociodemographic and clinical characteristics and VEGF of participants

	RS	NRS	HC	Statistic
	(*n* = 65)	(*n* = 45)	(*n* = 58)	
Age (years)	29.3 ± 10.0	31.6 ± 13.4	31.4 ± 12.0	*F* = 0.698, *P* = 0.499
Male/Female	35/30	26/19	37/21	*χ*^2^ = 1.256, *P* = 0.534
Education (years)	10.8 ± 3.5	10.6 ± 3.6	11.8 ± 2.9	*F* = 1.861, *P* = 0.159
Smoker/non-smoker	38/27	25/20	32/26	*χ*^2^ = 0.160, *P* = 0.923
BMI (kg/m2)	25.0 ± 3.7	23.7 ± 4.6	24.3 ± 4.4	*F* = 1.335, *P* = 0.266
Age of onset (years)	25.9 ± 8.7	23.4 ± 8.6	/	*t* = 1.345, *P* = 0.234
Family history of mental disorders (yes/no)	11/54	11/34	/	*χ*^2^ = 0.940, *P* = 0.332
chlorpromazine equivalent	375.1 ± 130.5	478.8 ± 180.8		***t*** **= 3.298,** ***P*** **= 0.001**
VEGF (pg/mL)	422.8 ± 130.5	351.6 ± 121.8	438.6 ± 213.8	*F* =4.014 *P* = 0.020

NRS patients had low baseline VEGF levels (351.6 ± 121.8) in the serum compared to control (438.6 ± 213.8) and RS patients (422.8 ± 130.5) (*F* [2, 165] = 4.014, *P* = 0.020), as shown in Table 1. No significant correlations were found between serum VEGF levels and socio-demographic or clinical data (age of onset, duration of illness, and family history of mental disorders) in either all samples or within each group.

*RS* remitted first-episode schizophrenia; *NRS* non-remitted first episode schizophrenia; *HC* healthy control; *BMI* body mass index; *VEGF* vascular endothelial growth factor; Significant differences (*P* < 0.05) were marked in bold.

### Neurocognition in NRS, RS and HC groups

On nearly all neurocognitive tests (except for DST), RS performed significantly worse than HC but better than NRS (*P <* 0.05) (Table 2). These differences remained significant among the three groups after covarying for age, body mass index, education period, antipsychotic dose and combination therapy (*P* < 0.05).

**Table 2:** Comparison of neurocognition and social cognition among three groups

	RS	NRS	HC	F	P	Corrected P^a^	Corrected P^b^
	(*n* = 65)	(*n* = 45)	(*n* = 58)				
HVLT-R							
Total recall	22.9 ± 3.4	19.5 ± 2.3	27.0 ± 2.2	96.886	**0.000**	**0.000**	**0.000**
Delayed recall	8.9 ± 1.5	7.7 ± 1.6	10.7 ± 1.0	58.877	**0.000**	**0.000**	**0.000**
VFT-animals	13.5 ± 3.0	11.0 ± 2.7	14.9 ± 3.8	19.061	**0.000**	**0.000**	**0.049**
VFT-actions	10.3 ± 2.0	8.1 ± 2.5	10.8 ± 2.2	21.629	**0.000**	**0.000**	0.506
TMT-part A	63.9 ± 13.6	82.4 ± 8.3	47.0 ± 7.4	147.102	**0.000**	**0.000**	**0.000**
TMT-part B	147.2 ± 25.8	191.3 ± 24.0	118.2 ± 10.1	151.746	**0.000**	**0.000**	**0.000**
DST-Forward	8.0 ± 2.5	7.9 ± 1.9	8.2 ± 2.0	0.243	0.785	1.000	1.000
DST-Backward^c^	6.7 ± 1.7	6.3 ± 2.3	7.2 ± 2.1	2.838	0.061	1.000	0.374
Total	14.7 ± 2.9	14.2 ± 3.5	15.4 ± 3.8	1.712	0.184	1.000	0.746
Stroop words	93.4 ± 9.5	85.6 ± 8.2	101.9 ± 7.0	48.994	**0.000**	**0.000**	**0.000**
Stroop colors	64.6 ± 8.0	57.5 ± 8.6	71.2 ± 7.3	38.215	**0.000**	**0.000**	**0.000**
Stroopinterference^d^	37.7 ± 6.8	33.5 ± 7.7	43.7 ± 7.6	25.542	**0.000**	**0.013**	**0.000**
CFET							
Happy	19.7 ± 2.7	19.8 ± 2.2	20.1 ± 3.9	0.238	0.788	1.000	1.000
Sad	13.4 ± 2.8	8.9 ± 2.5	13.3 ± 2.8	43.686	**0.000**	**0.000**	1.000
Fear	6.1 ± 2.2	4.0 ± 2.1	6.8 ± 2.2	21.404	**0.000**	**0.000**	0.242
Disgust	11.8 ± 2.5	10.5 ± 2.1	12.7 ± 2.2	11.235	**0.000**	**0.011**	0.129
Angry	12.7 ± 2.5	9.8 ± 3.1	13.0 ± 4.0	14.467	**0.000**	**0.000**	1.000
Surprised	9.9 ± 1.9	10.0 ± 2.9	11.0 ± 3.0	3.561	0.076	1.000	0.051

Abbreviations: *RS* remitted first-episode schizophrenia; *NRS* non-remitted first episode schizophrenia; *HC* healthy control; *HVLT-R* Hopkins Verbal Learning Test-Revised; *VFT* verbal fluency tests; *TMT* trail making tests; *DST* digit span tests; *CFET* Chinese Facial Emotion Test; Significant differences (*P* < 0.05) were marked in bold

^a^Refers to Bonferroni corrections of the comparisons between RS and NRS groups

^b^Refers to Bonferroni corrections of the comparisons between RS and HC groups

^c^NRS patients *n* = 44

^d^NRS patients *n* = 44

### Social cognition in NRS, RS and HC groups

In contrast to RS, NRS performed markedly poorer on all social cognition tests than controls (*P* < 0.05), with the exception of the Happiness and Surprise subscales of the CFET (Table 2), that is, correct identification of negative emotions (sadness, fear, disgust and anger) of CFET was lower in NRS than in RS (*P* < 0.05); the latter group did not differ from controls. This difference remained significant after covarying for antipsychotic dose.

### Correlation among VEGF, neurocognition and social cognition performance

**Fig. 1:**
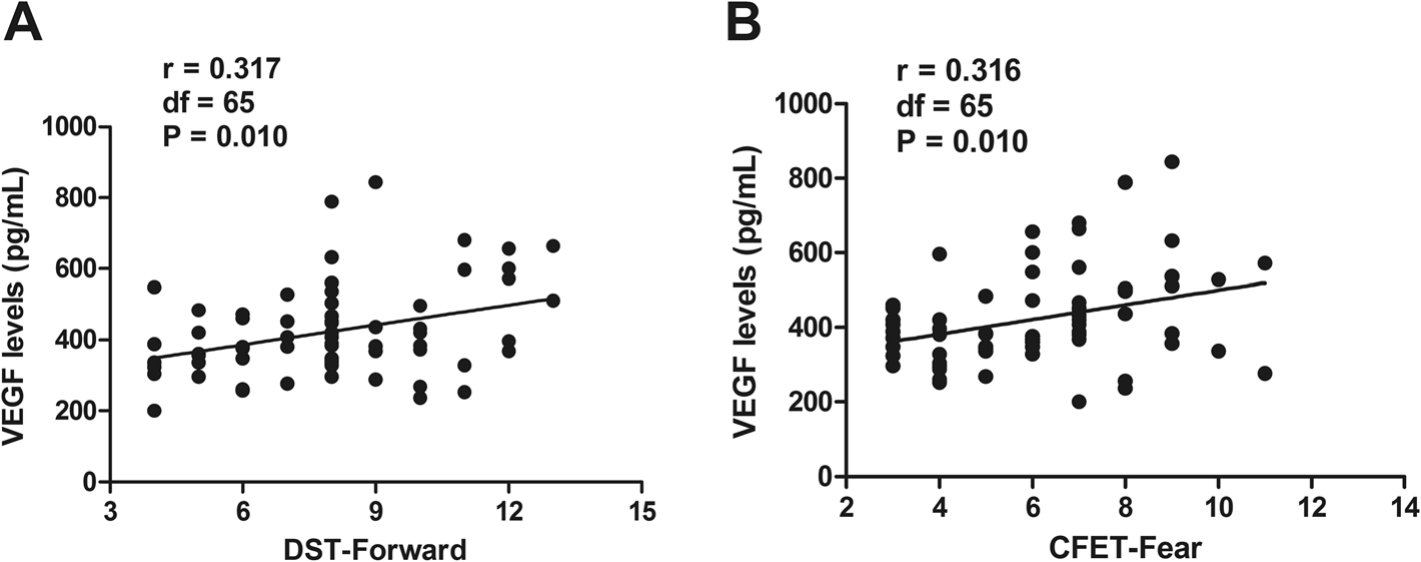
(A) Correlation between VEGF and DST-Forward in RS. (B) Correlation between VEGF and CFET-Fear in RS. VEGF = vascular endothelial growth factor; DST = digit span tests; CFET = Chinese Facial Emotion Test

Serum VEGF levels showed a significant positive association with TMT-part B (*r* = 0.265, *df* = 58 *P* = 0.044) and CFET-Anger (*r* = 0.270, *df* = 58, *P* = 0.040) in HC. Additionally, VEGF levels were positively correlated to DST-Forward (*r* = 0.317, *df* = 65, *P* = 0.010) and CFET-Fear (*r* = 0.316, *df* = 65, *P* = 0.010) in RS (Table 3, Fig. 1). After controlling for demographic and psychopathological parameters using partial correlation analysis, the relationship between VEGF, neurocognition and social cognition performance was statistically significant only in RS, but not in HC. However, for NRS patients, no significant association between VEGF and any cognitive domain subscore was observed (all *P* > 0.05). Our results show a gradient among deficits in cognition across the groups (NRS > RS > HC) and VEGF levels among them also turns out that they share the different changing trend(HC > RS > NRS).

**Table 3:** Association between VEGF, neurocognition and social cognition performance

	RS (*n* = 65)		NRS (*n* = 45)		HC (*n* = 58)	
	*r*	*P*	*r*	*P*	*r*	*P*
HVLT-R						
Total recall	−0.223	0.075	− 0.025	0.873	− 0.102	0.447
Delayed recall	−0.040	0.753	−0.049	0.749	−0.118	0.379
VFT-animals	−0.057	0.649	0.025	0.868	1.000	0.454
VFT-actions	0.067	0.594	−0.160	0.293	−0.165	0.216
TMT-part A	0.165	0.189	0.077	0.616	0.170	0.201
TMT-part B	−0.054	0.672	−0.096	0.532	0.265	**0.044**
DST-Forward	0.317	**0.010**	−0.019	0.903	0.133	0.320
DST-Backward	−0.075	0.552	−0.020	0.897	−0.101	0.452
Total	−0.085	0.500	0.008	0.985	0.051	0.703
Stroop words	0.235	0.060	0.131	0.390	0.007	0.985
Stroop colors	0.028	0.825	0.042	0.785	0.127	0.343
Stroop interference	−0.131	0.298	0.089	0.562	−0.094	0.482
CFET						
Happy	−0.022	0.862	−0.102	0.506	0.133	0.321
Sad	−0.063	0.619	−0.160	0.295	−0.095	0.480
Fear	0.316	**0.010**	0.072	0.636	−0.194	0.145
Disgust	−0.130	0.301	0.153	0.317	−0.049	0.713
Angry	−0.235	0.060	0.127	0.407	0.270	**0.040**
Surprised	0.238	0.056	0.110	0.473	0.109	0.414

Abbreviations: *RS* remitted first-episode schizophrenia; *NRS* non-remitted first episode schizophrenia; *HC* healthy control; *HVLT-R* Hopkins Verbal Learning Test-Revised; *VFT* verbal fluency tests; *TMT* trail making tests; *DST* digit span tests; *CFET* Chinese Facial Emotion Test. Significant differences (*P* < 0.05) were marked in bold

## Discussion

To our knowledge, no published studies have simultaneously examined neurocognition, social cognition (facial emotion recognition) and VEGF levels in RS and NRS and HC. First of all, our results showed that RS have poorer neurocognitive function than HC, but perform better than NRS. This is in agreement with prior studies [8] demonstrating that neurocognition was impaired in RS even if the patient’s psychiatric symptoms were relieved. Moreover, Boden et al. [26] found that RS had better social behavior and subjective life satisfaction than NRS. In addition, Debrecen scholars have demonstrated cognitive impairment in the remission stage of patients with schizophrenia, suggesting that these deficits might be permanent [27, 28].

Interestingly, we also found that identification of the correct number of negative emotions (sadness, fear, anger, and disgust) was significantly lower in NRS than HC, while RS were not different from HC. This observation is consistent with previous findings [8, 29, 30] and may be explained by residual psychotic symptoms (e.g. paranoia and delusions) in NRS. Another possible explanation may be the effect of antipsychotic drugs on social cognition. Similarly, Balogh et al. [31, 32] suggested that social cognition dysfunctions are associated with acute episodes, particularly with positive psychiatric symptoms, and that these impairments are temporary. However, there is also inconsistent evidence. For example, Rodríguez-Sosa et al. [33] reported that social cognition deficit was still present in discharged patients with stable condition. These differences may be related to the heterogeneity of the sample (the unlike genetic background of different ethnicities), first-episode or relapse, anti-psychotic drug dose or illness severity. Most important of all, we included first-episode schizophrenic patients in the active stage or in clinical remission, which is our major difference. In short, our findings support impaired social cognition performance as a state of disease activity in schizophrenia. Further studies using larger sample sizes are needed to draw definitive conclusions.

In the current study, patients with first-episode schizophrenia presented with markedly impaired neurocognitive function, despite being in symptomatic remission. These results were different from social cognition measured by facial emotion recognition. However, some authors have pointed out that social cognition is associated with neurocognition [12, 34]. Conversely, Mehta et al. [35] investigated the specificity and severity of social cognition deficit in schizophrenic patients and found that Theory of Mind was not affected by cognitive ability. Therefore, social cognition and neurocognition may have distinct mechanisms.

Our most important findings are that VEGF levels are decreased in NRS patients compared to RS and HC. Furthermore, we previously reported a decrease in the VEGF in serum of subjects with schizophrenia that is restored in schizophrenia subjects treated with antipsychotic drugs or ECT [36–38]. These results are consistent with previous studies [39–44], supporting the hypothesis that levels of neurotrophic factor of the patients with schizophrenia was obviously abnormal. In addition, we found that VEGF was more strongly associated with neurocognitive deficits and social functioning in RS rather than NRS. However, the mechanism underlying this association remains unclear. There is compelling evidence VEGF may limit cognitive impairment, reduce dendritic spine loss and protect the blood-brain barrier [14, 17, 45–47]. Recent experimental evidence suggests that VEGF ameliorates cognitive and emotional deficits in an animal model of type 2 diabetes [48]. VEGF improves recovery of cognitive deficits by mediating the effect of hippocampal neurogenesis [49, 50], which links hippocampal activity with neurogenesis, learning and memory [16]. In addition, VEGF has been shown to exert robust neuroprotective effects by improving cognitive abilities in mice [51], and revert the cognitive impairment induced by a focal traumatic brain injury during the development of rats raised under environmental enrichment [52]. Human studies have found that VEGF levels were also associated with cognitive function in patients with Alzheimer’s disease [15, 19, 20]. VEGF may ameliorate cognitive impairment via improving neuronal viability and function [53], which may explain the relationship between cognitive function and VEGF in individuals with remitted schizophrenia.

### Limitations

This study has some limitations. First, the sample size was relatively small. Second, the antipsychotic dose differed between RS and NRS patients. A potential effect of antipsychotic drugs on cognition cannot be excluded, although a meta-analysis showed that the second generation of antipsychotic drugs does not affect cognition in schizophrenia [54]. Third, our cross-sectional design may affect the precision and replication of the results. Finally, we only assessed one aspect of social cognition (facial emotion recognition), while other areas (psychological theory and attribution) were not evaluated. Notwithstanding these limitations, this study does suggest that VEGF is related to neurocognition and facial emotion recognition functioning in RS. Future clinical trials with additional neurocognition and social cognition tests are required to confirm our results.

## Conclusions

Our study showed that remitted first-episode schizophrenic patients had deficits in neurocognition but not social cognition, and that both were associated with VEGF serum levels. With the severity of cognitive impairments in schizophrenia, VEGF levels decreased gradually. Interestingly, VEGF may be involved in the mechanisms underlying cognitive function in remitted patients, although the mechanism about the association remains poorly understood. VEGF may serve as a sensitive monitor to estimate the degree of cognitive impairments and clinical prognosis in schizophrenia.

## Data Availability

The data that support the findings of this study contain sensitive personal information and thus are not publicly available as they are subject to secrecy. The datasets used during the current study are available from the corresponding author on reasonable request.

## Abbreviations

CFET: Chinese Facial Emotion Test
DST: Digit span tests
HC: Healthy control
HVLT-R: Hopkins Verbal Learning Test-Revised
NRS: Non-remitted first episode schizophrenia
RS: Remitted first-episode schizophrenia
TMT: Trail making tests
VFT: Verbal fluency tests
